# Treatment of Gastrointestinal Sphincters Spasms with Botulinum Toxin A

**DOI:** 10.3390/toxins7061882

**Published:** 2015-05-29

**Authors:** Giuseppe Brisinda, Nicola Sivestrini, Giuseppe Bianco, Giorgio Maria

**Affiliations:** Department of Surgery, University Hospital “Agostino Gemelli”, Largo Agostino Gemelli 8, 00168 Rome, Italy; E-Mails: nicola_silvestrini@libero.it (N.S.); bianco.g@live.com (G.B.); giorgio.maria@rm.unicatt.it (G.M.)

**Keywords:** anus, physiopathology, autonomic nervous system diseases, biliary diseases, botulinum toxin, therapeutic use, chronic constipation, enteric nervous system, esophageal achalasia, esophageal diseases, exocytosis, fissure-in-ano, gastric emptying, gastrointestinal motility, membrane fusion, membrane proteins, neuromuscular agents, obesity, pain, spasm

## Abstract

Botulinum toxin A inhibits neuromuscular transmission. It has become a drug with many indications. The range of clinical applications has grown to encompass several neurological and non-neurological conditions. One of the most recent achievements in the field is the observation that botulinum toxin A provides benefit in diseases of the gastrointestinal tract. Although toxin blocks cholinergic nerve endings in the autonomic nervous system, it has also been shown that it does not block non-adrenergic non-cholinergic responses mediated by nitric oxide. This has promoted further interest in using botulinum toxin A as a treatment for overactive smooth muscles and sphincters. The introduction of this therapy has made the treatment of several clinical conditions easier, in the outpatient setting, at a lower cost and without permanent complications. This review presents current data on the use of botulinum toxin A in the treatment of pathological conditions of the gastrointestinal tract.

## 1. Introduction

Although the therapeutic potential of botulinum toxin (BT) for skeletal muscle disorders was first realized in the 1970s [1], it was not until nearly two decades later that it was also shown to be effective in the gastrointestinal tract (GIT) [2,3,4,5]. Since then, however, there has been a rapid increase in the number of reports in a variety of GIT conditions characterized by dysfunctional smooth muscle [5,6]. This article will review the BT efficacy in the treatment of these conditions. In particular, we have taken into account all applications at all level of the GIT. We have reported the latest findings of the literature on the BT use in the treatment of GIT diseases. We believe that the introduction of BT injection in the treatment of these patients represents an innovation equal to the introduction of laparoscopy. The introduction of this therapy has made the treatment of several clinical conditions easier, in the outpatient setting, at a lower cost and without permanent complications.

## 2. Background

Normal GIT motility depends on intrinsic neurons contained in the enteric nervous system (ENS), with significant modulatory input being provided by the central nervous system (CNS) via autonomic sympathetic and parasympathetic nerves [7,8]. Immediate control of muscle tone in the gut reflects a balance between both excitatory (predominantly cholinergic) and inhibitory (predominantly nitrinergic). In some disease states, this balance is disrupted, usually due to a relatively selective loss of inhibitory neurons [9,10,11]. In this setting, BT, by blocking excitatory neurotransmitter release, can restore the balance and cause a decrease in the resting tone of the muscle involved.

Although BT can clearly inhibit the release of acetylcholine, little else is known about its effects in GIT muscle. Thus, while nitric oxide (NO) release is not affected—which is to be expected, since this is not a vesicular process—the specific effects on other potentially important neurotransmitters has not been well documented [12,13]. Further, there is some suggestion that it may also inhibit the responsiveness of smooth muscle to exogenous stimuli, an effect that is quite unique to the GIT.

GIT smooth muscle has an inherent motility and coordinated, aimed at the progression of ingested food from the oropharynx to the anal canal. The different regions of the gastrointestinal tract motility own and have a characteristic, capable, however, each play a specific role. The term includes motility, in fact, a number of events summarized as follows:

1. Ability of smooth muscle cells to contract (myogenic event);
2. Coordination of the contraction of smooth muscles, nerves through intrinsic and extrinsic (neurogenic event);
3. Coordinated muscle contraction, with subsequent increase of the intraluminal pressure;
4. Propulsion of content, which is the final event with the participation of the above.

All these functions are under the direct control of the so-called ENS. It consists of all the neurons of the gastrointestinal tract and shows an independent function of the central nervous system, so as to merit the label “brain of the gut”.

A deficiency of enteric neurons causes obstruction and lack of intestinal propulsion [14]. The ENS is composed of two main ganglionated plexuses (Auerbach’s myenteric plexus and Meissner’s sub-mucous plexus) and non-ganglionated plexuses (the longitudinal muscle plexus, the circular muscle plexus, the plexus of the muscularis mucosae, and the mucosal plexus) [8]. Intraparietal neurons encompass motor excitatory and inhibitory neurons, interneurons and intrinsic sensory neurons. Sympathetic and parasympathetic neurons also innervate the GIT. The primary excitatory transmitter is ACh, while inhibitory transmitters are NO, adenosine triphosphate (ATP), and VIP [8].

At the cellular level, smooth muscle contraction and relaxation are regulated by changes in cytosol calcium levels [15]. These functions depend on the intrinsic electrical and mechanical properties of GIT smooth muscles and are regulated by the ENS and by sympathetic and parasympathetic influences [8]. Hormones also influence GIT motility [16]. Interstitial cells of Cajal act as local pacemakers to generate the rhythmic activity of the circular muscle layer throughout the GIT. Motor neurons control the musculature indirectly, through their action on Cajal’s cells. Substances, such as histamine, serotonin, adenosine, and eicosanoids, produced by non-neural cells, can influence smooth muscle activity [17].

## 3. Esophageal Applications

### 3.1. Cricopharyngeal Dysphagia

Dysphagia associated with cricopharingeal (CP) muscle dysfunction has a significant impact on overall patient quality of life [18]. CP dysphagia, either idiopathic or secondary to various neurologic or muscular conditions, is characterized by incomplete or poorly coordinated opening of the upper esophageal sphincter (UES) during swallowing. This proximal dysphagia can result in laryngeal penetration or tracheal aspiration of swallowed food. The CP muscle is therefore often a target of surgical interventions for dysphagia, including bougie or balloon dilatation, myotomy and chemodenervation by treatment with BT [18,19,20,21,22,23]. UES dilation is often effective and of low risk, but also has a short clinical effect. Transcervical CP myotomy is effective in treating CP dysphagia, but has significant risk of infection, salivary fistula formation, and recurrent laryngeal nerve injury [24]. Endoscopic laser CP myotomy was described in the early 1990s, with evidence of successful treatment in multiple patient series. CP myotomy improves UES opening, but will not alter pharyngeal muscle contractile forces, and therefore may not benefit every patient with CP dysphagia. Traditionally, CP myotomy has been the mainstay of treatment but other options such as dilation and BT injection have been used with good results [18,23]. BT injection into the CP muscle to treat dysphagia was first described in 1994 by Schneider and co-workers, [25] in a series of 7 patients, as an alternative treatment to the more invasive myotomy procedures.

A number of injection techniques have been employed including rigid endoscopy with electromyographic control, flexible endoscopy, and an open technique with various doses (10–50 units). Endoscopically, 3 to 4 injections of BT can be delivered to the dorsomedial and bilateral ventromedial compartments of CP muscle. Furthermore, the location of the CP muscle has been verified by EMG in a number of studies especially in the otolaryngology literature.

CP injection of BT has distinct appeal in patients who are not ideal candidates for longer general anesthesia or in whom the temporary nature of BT injection is warranted. It may be advantageous to pursue CP injection of BT in patients in whom multilevel dysphagia is suspected and in whom the clinician suspects that there may be some detriment to treatment directed at the UES. Additionally, CP injection of BT is a diagnostic tools used by clinicians to identify patients who may potentially benefit from CP myotomy [18,20,21,26]. A review of the literature identified 20 studies that focused on the use of CP injection of BT (Table 1).

**Table 1:** Review of the literature on the treatment of crycopharingeal dysphagia with BT injection.

Authors	Pts	Botox (Unit)	Dysport (Unit)	Improvement	Method of Delivery	Causes	Complications
Schneider *et al.*, 1994 [25]	7	80–120		5/7 (71%)	GA, EGD	Stroke, CN palsies, supraglottic or oropharyngeal cancer, reflux disease	None
Atkinson and Rees, 1997 [27]	5	5–20		4/5 (80%)	CT guided injection	Stroke, CN palsies, bulbar palsy	Left vocal fold paresis, aspiration pneumonia
Blitzer and Brin, 1997 [26]	6	10		6/6 (100%)	Percutaneous injection	CVA, partial pharyngectomy, small Zenker’s diverticulum	None
Alberty *et al.*, 2000 [28]	10	30		10/10 (100%)	GA, EGD	CVA, idiopathic polymiositis	None
Shaw and Searl, 2001 [29]	12	25–50		10/12 (83%)	GA, EGD, open technique	Progressive neuropathy, oculopharyngeal dysphagia, skull base tumor resection, total laryngectomy, CVA, partial pharyngectomy, CNS neuropathy	Pharyngeal tear, worsening dysphagia
Haapaniemi *et al.*, 2001 [30]	4	14–50		3/4 (75%)	GA, EGD	Brain stem stroke, inclusion body myositis,peripheral motor neuropathy, CVA	None
Moerman *et al.*, 2002 [31]	4	100		4/4 (100%)	GA	Head and neck cancer resection including total laryngectomy, radiation	None
Parameswaran and Soliman, 2002 [32]	12	10–30		11/12 (92%)	EGD with mask ventilation and apneic technique	Idiopathic, radiation, CVA, total laryngectomy, ALS, Parkinson’s disease	Neck cellulitis (concurrent thyroglossal duct excision)
Zaninotto *et al.*, 2004 [33]	21	4–10		9/21 (43%)	Percutaneous with EMG	CNS disease, peripheral neuropathies, idiopathic	Death of aspiration (attributed to underlying disease)
Murry *et al.*, 2005 [34]	13	100		11/13 (85%) 2/13 improvement after second injection	EMG-guided transcutaneous approach	Stroke, head and neck surgery, cranial neuropathies, MVC, chemical inhalation, radiation therapy or lymphoma	None
Kim *et al.*, 2006 [35]	8	100		5/8 (62.5%)	Flexible endoscopy	CVA	None
Restivo *et al.*, 2006 [36]	12		60	12/12 (100%)	EMG-guided transcutaneous approach	Diabetic neuropathy	None
Alfonsi *et al.*, 2010 [37]	34	15		17/34 (50%)	EMG-guided transcutaneous approach	MS, Multiple system atrophy, Parkinson’s disease, progressive sopranuclear palsy, ataxia-teleangectasia	None
Restivo *et al.*, 2011 [38]	14	20		14/14 (100%)	Percutaneous injection with EMG guidance	MS	None

ALS, amyotrophic lateral scrlerosis; CN, cranial nerve; CNS, central nervous system; CT, computed tomography; CVA, cerebrovascular accident or stroke; EGD, esophagogastroduodenoscopy; EMG, electomyography; GA, general anesthesia; MVC, motor vehicle collision; SAH, subarachnoid hemorrhage; MS, multiple sclerosis.

Only two series were of more than 20 patients; the largest study included 34 patients. The causes of CP dysfunction in these published series encompassed several diagnosis, including neurological diseases, diabetic neuropathy, external-beam radiation treatment, cerebrovascular accident, and others. The dosage and administration techniques of BT were also quite variable [18]. There were also different types of BT administered: Dysport (Ipsen, Paris, France) and Botox (Allergan, Irvine, USA); the Dysport doses delivered to CP muscle ranged from 60 to 180 units, and the Botox doses ranged from 4 to 120 units [18,39].

In general, the majority of patients reported improved swallowing function: approximately 75% in combined analysis. Complications were infrequent and included transient vocal fold paresis, temporary worsening of dysphagia, neck cellulitis, and aspiration pneumonia. There were no reported deaths in the literature that were directly related to CP injection of BT. Recently, Kelly and coworkers demonstrated that CP injection of BT is a well-tolerated treatment for dysphagia related to CP dysfunction, with good efficacy in the majority of their 49 patients [18]. On the basis of these results, CP injection of BT appears to be effective in patients with UES dysfunction. Response to BT injection may select out a group of patients with higher likelihood of a more durable response to surgical myotomy [40]. Further work, however, is needed to define the population of patients who might have a poor response to BT treatment. Furthermore, non-response may indicate another etiology of dysphagia, *i.e.*, stricture.

#### Crycopharingeal Achalasia (CPA) in Children

The condition is characterized by an incomplete relaxation or by a lack of coordination of the UES [22,41]. CPA is a different entity then the CP dysphagia that was see in adults. The exact cause of CPA is unknown. Immaturity of the interstitial cells of Cajal may explain why there have been reports of spontaneous resolution of CPA seen in infants [42]. CPA has also associated with gastro-esophageal reflux disease and CNS abnormalities [21,22,41,42,43,44].

Recently, six children were identified with CPA [22]. The decision to proceed with BT therapy was based on ongoing severe symptoms, the necessity of altered feeds, and parent preference over a surgical myotomy. The number of injections ranged from 1 to 3 per patients. The mean dose was 5.6 units/kg, with a range of 1.6 units/kg to 7.9 units/kg and a median of 6.0 units/kg. In those patients with multiple injections, the mean time between injections was approximately 13 months. The mean time to return to normal radiographic swallow study was 8.2 weeks. Two of the children benefited from BT injections and went on to have CP myotomy, while four of the children did not require myotomy and their symptoms resolved after one or two injections. The authors concluded that BT injection of CP muscle is a useful tool to help diagnose and treat CPA [22]. It is a feasible alternative to more invasive surgical procedures. However, more research is needed to elucidate the optimal dosing, frequency of injections, and when to move on to surgical intervention.

### 3.2. Achalasia

The major pathophysiological lesion in achalasia, which means failure to relax, results from a relatively specific loss of nitrergic inhibitory neurons of the LES, resulting in an inability of the sphincter to relax after swallowing [45]. This results in a functional obstruction and dysphagia. Although no cure exists for achalasia, there are a number of palliative treatments available including surgical myotomy, pneumatic dilation (PD), and BT injections into the LES [46,47,48,49,50,51]. Surgical myotomy has proven durable, but is associated with increased morbidity and mortality in high-risk surgical patients. Pneumatic dilation of the sphincter results in an initial symptomatic improvement in 60%–90% of patients but repeated dilations are often necessary. Furthermore, the procedure carries a small but significant risk of esophageal perforation [52,53,54]. Thus, BT provides a potentially attractive alternative to the above treatment methods [49].

Endoscopic injection of 25 units of BT in 4 LES quadrants is generally the standard of care. The efficacy of BT in achalasia has been proven by the results of several randomized trials comparing it to either placebo or pneumatic dilation. Table 2 summarizes the response rates to BT in patients with achalasia.

In general, 75%–100% of patients show an initial response but more sustained improvement (beyond 6 months) is seen in about two-thirds. For unclear reasons, it appears that patients older than 50 years of age respond at a higher rate (82% *vs.* 43% in younger patients). Similarly, patients with so-called vigorous achalasia (with the esophagus retaining some contractile ability) respond at a higher rate (100% *vs.* 52% with classic achalasia).

**Table 2:** Review of experiences using BT for the treatment of esophageal achalasia.

Authors	Description	Patients	Results/Conclusions
Pasricha *et al.*, 1995 [5]	BT *vs.* Placebo	21	67% improvement at 6 weeks
Annese *et al.*, 1996 [55]	BT *vs.* placebo *vs.* PBD	16	100% improvement at 1 month. BT is as effective as pneumatic dilatation
Fiorini *et al.*, 1996 [56]	BT *vs.* Placebo	13	72% improvement at 3 month
Pasricha *et al.*, 1996 [57]	BT	31	60% (82% of those aged > 50) improvement at 3 month
Fishman *et al.*, 1996 [58]	BT	65	60 idiopatic cases: BT treatment improved symptoms of dysphagia, Chet pain and regurgitation in the majority of patients. 5 secondary cases: There was no response to BT in 4 patients. Patients, who respond to a first BT injection but relapse, may respond to a second treatment
Cuilliere *et al.*, 1997 [59]	BT	55	60% improvement at 6 month
Kolbasnik *et al.*, 1999 [60]	BT	30	Symptomatic improvement for >3 month was seen in 77% of patient. 7 patient had a sustained response after a single injection; 16 relapsed and required re-treatment
Annese *et al.*, 1999 [61]	Botox *vs.* Dysport	78	Comparable efficacy in esophageal achalasia after up to 6 month after treatment
Muehldorfer *et al.*, 1999 [62]	BT *vs.* PBD	24	The two treatment had equal initial success rate (dilatation 83%, BT 75%). In the long term the efficacy of BT injection was statistically significantly and shorter than that of balloon dilatation
Greaves *et al.*, 1999 [63]	BT	11	The relapse rate was 73% within 2 years from treatment. There were a beneficial effect on dysphagia, no improvement in chest pain or regurgitation scores, and no reduction of mean LES pressure were improved at 6 weeks
Wehrmann *et al.*, 1999 [64]	BT in high risk patients	20	80% were improved at 6 weeks. Mean cardia diameter was increased from 2.1 mm to 3.2 mm. The patients who initially had a symptomatic relapse after an average of 5 months. BT re-injections were efficacious
Hurwitz *et al.*, 2000 [65]	BT in children	23	The mean duration of effect in 19 responders was 4.2 months. 50% of the patients required an additional procedure (PD, surgery) on average 7 months after the first treatment
Annese *et al.*, 2000 [66]	BT dose raging study	118	82% of the patients were responders at 1 month. No dose related effect was observed. Vigorous achalasia was the main determinant of BT response
Mikaeli *et al.*, 2001 [67]	BT *vs.* PBD	40	Cumulative 12-month remission rate was significantly higher after a single PD (53%) compared to a single BT injection (15%, *p* < 0.01). The 12-month estimated adjusted hazard for relapse and need for retreatment for BT group was 2.69 times that of the PD group
Allescher *et al.*, 2001 [68]	BT *vs.* PBD	37	After 24 months a single PD was superior to a single BT injection, and after 48 months all patients treated for BT injection had experienced a symptomatic relapse
Ghoshal *et al.*, 2001 [69]	BT *vs.* PBD	17	Both therapies resulted in a significant reduction in LES pressure
Zarate *et al.*, 2002 [70]	BT	17	The effect of BT injection wanes with time in elderly patients, necessitating repeated injections to keep the patients symptoms free
D’Onofrio *et al.*, 2002 [71]	BT	37	Of the 35 patients followed, 12 had a relapse and were treated; 4 out of 12 did not respond after treatment. One or two BT injections result in a clinical and objective improvement in about 84% of achalasia patients and are not associated with serious side-effects; patients over 50 years showed better benefit than younger patients
Neubrand *et al.*, 2002 [72]	BT	25	Good results after 2.5 years of median follow up in 9 of 25 patient that were significantly older than 14 patients for whom BT treatment was unsuccessful
Brant *et al.*, 2003 [73]	BT in Chagas’ disease	24	Over a period of 6 month, clinical improvement of dysphagia was statistically significant (*p* < 0.001) in patients receiving BT when compared with the placebo. Esophageal emptying time in BT group was significantly lower than in the placebo (*p* = 0.04) after 90 days
Bansal *et al.*, 2003 [74]	BT *vs.* PBD	32	After 12 month follow up 16 of 18 patients of PBD were in clinical remission despite 6 of 16 of BT group
Martinek *et al.*, 2003 [75]	BT *vs.* PBD	41	16 patients had BT injection from the antegrade angle only (group A), 15 both from antegrade than retrograde (group B) and 10 had subsequent PD (group C). 93% had an immediate clinical response after 1 month ad 49 were in remission after 22 months. Better responders were older and with lower LES pressure. Patients in group C had better results at 1 and 2 year
Vela *et al.*, 2004 [76]	PBD *vs.* HM *vs.* BT	232	111 patients underwent PBD, 72 HM and 39 elderly patients BT injection. 48 patients had already surgical treatment and underwent to PBD or redo-HM. PBD and HM are the best treatments for untreated achalasia and are less successful after surgery. BT group needed repeated injections and their symptoms improving lasted for a mean period of 6.2 months
	PBD *vs.* HM in patient with prior surgery		
Zaninotto *et al.*, 2004 [77]	BT *vs.* HM	80	After 6 months similar results were reported in the 2 groups of 40 patients, but after 2 years 87.5% of patients of surgical groups were symptoms free *vs.* 34% of BT group (*p* < 0.05)
Mikaeli *et al.*, 2004 [78]	BT + PBD *vs.* PBD	24	BT + PBD (case-group) had a significant higher cumulative remission rate compared to control (PBD) group (24.6 *vs.* 12.6 months *P* < 0.01) and a significant reduction in symptom-score (76% *vs.* 53% *P* < 0.001). Control group needed a 35 mm PBD *vs.* 30 mm of case group
Dughera *et al.*, 2005 [79]	BT elderly	12	After 12 months of follow-up, up to 70% of patients were considered responders. They underwent 2 BT injection (time 0 and after 1 month). Average age 86 y.o. ASA 3 or 4
Bassotti *et al.*, 2006 [80]	BT elderly	33	Patients underwent 2 BT injection (time 0 and after 1 month). 78% were considered responders after 1 year and 54% after 2 years. No relationship was found between baseline LES pressure and symptoms score
Mikaeli *et al.*, 2006 [81]	BT + PBD *vs.* PBD	54	77% of patients of BT + PBD group were in remission after 1 year *vs.* 62% of PBD group and showed a significant reduction in barium volume at the various times intervals post-treatment
Zhu *et al.*, 2009 [82]	BT *vs.* PBD *vs.* BT + PBD	90	LES pressure and symptom score in group C (BT + PBD) were significantly lower compared with those in group A (BT) or group B (PBD) (*p* < 0.05). At 2 years after treatment, the response rate in group C remained 56.67% *vs.* 35.71% (group B) and 13.79% (group A) (*p* < 0.05)
Kroupa *et al.*, 2010 [83]	BT + PBD *vs.* PBD	91	The mean duration of follow-up was 48 months (12–96 months). 41 of 51 patients were followed up more than 2 years. Effect of therapy lasted in 75% (31/41) of them. The cumulative 5 years remission rate in combined treated patients was higher than in controls bu not statistically significant. (*p* = 0.07). Injection of BT followed by PD seems to be effective for long-term result but the combined therapy is not significantly superior to PD alone
Gutschow *et al.*, 2010 [84]	BT *vs.* PBD *vs.* PBD-HM *vs.* HM	41	Patients of BT group (*n* = 7) had the lower mean LES pressure (18.1 mm Hg) and higher recurrence rate (71.4%) compared to patients of PBD group (*n* = 16, 34.8 mm Hg—50%), PBD-HM group (*n* = 14, 22.2 mm Hg—35.7%) and HM group (*n* = 6, 36.4 mm Hg—16.7%)
Bakhshipour *et al.*, 2010 [85]	BT + PBD *vs.* PBD	34	Patient of study-group already underwent two initial PBD with a low response. They were randomized to receive another PBD or BT injection and PBD by four weeks interval. BT + PBD group had higher remission rate at 1, 6 and 12 months compared to PBD group (87.5% *vs.* 67.1%, 87.5% *vs.* 61.1%, 87.5% *vs.* 55.5%, respectively). Difference was not statistically significant
Porter *et al.*, 2011 [86]	BT	36	Response lasted a mean of 12.8 months and symptom relief for > 6 months was seen in 58.3% of patients. Chest pain, younger age and contraction amplitudes >180 mmHg independently predicted <6 months relief (*p* < 0.05 for each)
Ciulla *et al.*, 2013 [87]	BT	68	36 patients underwent echo-guided BT injection had complete relief of obstruction compared to 32 patients who underwent blind treatment.
Cai *et al.*, 2013 [88]	BT *vs.* SEMS	110	Improvements in global symptom, dysphagia scores and in LES pressure were significantly more marked in the SEMS group (*n* = 59) than in the BT group (*n* = 51). Remission rate in the SEMS group was statistically significantly higher than that in the BT group at 12 and 36 months [81.28 *vs.* 64.58 (*p* < 0.05) and 49.1 *vs.* 4.2 (*p* < 0.01)]. No side effects were reported in BT group *vs.* 26 in SEMS group
Jung *et al.*, 2014 [53]	BT *vs.* PBD	37	A significant difference was observed in the mean remission duration between the BT injection (*n* = 25) and PBD (*n* = 12) (13 months *vs.* 29 months). Independent factors predicting long-term remission included treatment type and the difference in the initial LES pressure
Marjoux *et al.*, 2014 [48]	BT	45	22 patients had achalasia, 8 jackhammer esophagus, 7 distal esophageal spasm, 5 esophagogastric junction outflow obstruction, 1 nutcracker esophagus, and 2 unclassified cases. 71% were significantly improved after 2 months and 57% remained satisfied for more than 6 months. No clear difference was observed in terms of response according to manometric diagnosis. Type 3 achalasia had the worst outcome with none of these patients responded to the endoscopic BT injection

BT, Botulinum toxin; HM, Heller myotomy; LES, lower esophageal sphincter; PBD, pneumatic balloon dilatation; PD, pneumatic dilatation; SEMS, self-expanding metal stent.

Several studies have compared BT to pneumatic dilation with most reporting similar initial clinical or manometric responses. However, the one-year remission rate after a single injection is markedly inferior for BT, which is to be expected given its pharmacological properties. In the only study comparing the two modalities in a head to head comparison, 80 patients were randomized to receive 100 BT units or laparoscopic surgical myotomy with fundoplication. After six months, symptom scores improved more in surgical patients (82% *vs.* 66%, *p* < 0.05). The drop in LES pressure was similar in the two groups; the reduction in esophageal diameter was greater after surgery (19% *vs.* 5%, *p* < 0.05). The only complication in the surgical group was one patient bled at the trocar site. The probability of being symptom-free at two years was 87.5% after surgery and 34% after BT (*p* < 0.05). The same group investigated the cost effectiveness of the two modalities and concluded that BT was more cost effective in the short term, but at two years, cost between the two groups was similar. The results of a recent meta-analysis suggest that PD is the more effective endoscopic treatment in the long-term (greater than 6 months) for patients with achalasia [52].

BT injections into the upper GIT appear to be quite safe with very few, if any, reports of serious adverse effects. The incidence of gastro-esophageal reflux has not been well characterized in most studies but has been reported to be about 20%, by symptoms at least. There has also been some question in recent years whether BT prior to PD or myotomy complicates the more invasive procedures possible secondary to LES fibrosis. However, although previous BT injection (or PD for that matter) may make myotomy more challenging technically because of obliteration of tissue planes, this does not appear to affect the final outcome after myotomy.

### 3.3. Other Esophageal Disorders

BT has also been used in a variety of less well characterized esophageal conditions including diffuse esophageal spasm (DES) and patients with non-cardiac chest pain suspected to be on the basis of a dysfunctional esophagus. DES is a condition that is related to achalasia and may be associated with LES dysfunction as well [89,90,91,92,93,94,95,96]. The largest clinical trial assessing the effect of BT in DES patients evaluated 9 patients [97,98]. A significant reduction in symptom score was noted at week 4 and 8. A recent study examined 22 patients with DES or nutcracker esophagus who had primarily dysphagia and gave them blinded saline or BT injections in a crossover study design [94]. Results showed that symptoms scores and weight loss improved after BT treatment, not the saline injections, and this benefit was sustained for over a year in almost half of the patients. Unfortunately, there have been no other clinical trials evaluating BT as a treatment option for this disorder.

In addition to dysphagia and regurgitation, chest pain can be associated with achalasia, DES, ineffective esophageal motility (IEM), and isolated LES dysfunction which may respond to BT administration as shown in previous studies. A study, with improvement of chest pain as the primary end-point, evaluated 29 patients with non-cardiac chest pain who received 100 BT units injection into the LES, same as the treatment regimen for achalasia. Seventy-two percent of the patients responded with at least 50% reduction in chest pain [99].

Similar to idiopathic achalasia, in Chagas’ disease (CD), a common disease in South America [73,100,101,102,103,104], slow esophageal emptying is due to nonrelaxation of the LES. Only one representative series on 24 patients has been published regarding BT in esophageal CD. The authors have been showed that 58% of the patients had clinical improvement of dysphagia at 6 months follow up. Interestingly, gender, age and LES pressure did not influence outcomes, contrary to the results obtained in idiopathic achalasia series [57,73].

## 4. Gastric Applications

### 4.1. Gastroparesis

Gastroparesis or delayed gastric emptying resulting in nausea, vomiting, dyspepsia, and abdominal bloating is a common problem in patients seen by primary care physicians and gastroenterologists. Gastroparesis can occur as a result of poorly controlled diabetes mellitus, post-surgical manifestations, or idiopathic causes [105,106,107,108]. In recent years, BT injection into the pylorus has been investigated as a treatment option in this otherwise debilitating disorder.

The initial study evaluating the BT efficacy in patients with diabetic gastroparesis assessed six patients with abnormal solid phase gastric emptying studies [109]. Each patient received 100 BT units into the pyloric sphincter and symptom scores and gastric emptying were assessed after six weeks. There was an improvement of subjective symptom scores of 55%, which was maintained at six weeks. In addition, there was a 52% improvement in gastric emptying at six weeks. Another study investigated the BT use in cases of idiopathic gastroparesis [110]. Ten patients were given 80–100 BT units and a 38% reduction in symptom scores were seen at 4 weeks which correlated with findings of increased gastric emptying. A recent study evaluated the effects of BT on diabetic gastroparesis for 12 weeks [105]. Eight patients received 200 BT units into the pyloric sphincter, and seven patients completed the 12-week follow-up. Mean symptom scores declined from 27 to 12.1 (*p* < 0.01). Furthermore, six of the seven patients gained weight (*p* = 0.05) and gastric emptying scan time improved in four patients [105]. The largest study to address this issue retrospectively evaluated 63 patients who met the study criteria [108]. Gastroparesis was secondary to diabetes in 26 patients (41.2%), after surgery in two (3.2%), and idiopathic in 35 (55.6%). Twenty-seven of 63 (43%) patients experienced a symptomatic response to treatment (100 to 200 units) with a mean duration of five months. Male gender was associated with response to therapy. However, vomiting as a major symptom was predictive of no response to BT [108]. Further studies were needed to address these issues and to better define potentially responsive patients [111,112,113,114,115].

### 4.2. Obesity

BT injection into the gastric antrum may be used to transiently decrease gastric emptying as a treatment for obesity [116,117,118,119,120]. Preliminary data in rats have shown a significant loss of body weight associated with a reduction of dietary intake in the BT treated group. In a double blind controlled study, 24 morbidly obese patients (mean body mass index (BMI) 43.6 ± 1.09 kg/m^2^) were blindly randomized to receive 200 BT units or placebo into the antrum and fundus of the stomach by intraparietal endoscopic administration [121]. The two groups were homogenous for anthropometric characteristics. Eight weeks after the treatment, BT patients had significantly higher weight loss (11 ± 1.09 kg *vs.* 5.7 ± 1.1 kg, *p* < 0.001) and BMI reduction (4 ± 0.36 kg/m^2^ *vs.* 2 ± 0.58 kg/m^2^, *p* < 0.001) than controls. No significant side effects or neurophysiologic changes were found. Similar results have been found in an open label study of 10 obese adults (BMI 31–54 kg/m^2^) who received 100 units (4 patients) or 300 units (6 patients) of BT and were followed for 16 weeks [122].

Further results demonstrated that BT makes weight loss easier in obese patients [123]. It seems conceivable that BT acts by increasing the solid gastric emptying time and reducing the solid eating capacity of the stomach. However, the results in literature are controversial. In several clinical experiences, intragastric BT injection does not seems to reduce body weight [118,124,125,126].

### 4.3. Others Gastropyloric Disorders

BT has been used to facilitate gastric emptying in patients who underwent pylorus-preserving duodenopancreatectomy [127]. Initial studies suggests that BT injection into the pylorus improves both gastric emptying and symptoms.

Infantile hypertrophic pyloric stenosis is a congenital hereditary disorder characterized by a functional gastric outlet obstruction [128]. Obstruction is the result of a gradual hypertrophy of the circular smooth muscle of the pylorus, and the neurons that innervate the circular-muscle layer lack NO synthase. Recently it has been observed lack of response to BT injection in two patients with pyloric stenosis. Studies have shown that BT injection helps patients suffering from post-surgical pyloric clogging. BT injection is also used as an alternative method for the treatment of gastric emptying disorders [129,130,131]. In a recent study, the authors compared the effect of BT injection and pyloroplasty in preventing delayed gastric emptying after esophagectomy for esophageal cancer [115]. Sixty patients were included in the study: These patients were randomly divided into two groups. In group A, 30 patients underwent pyloroplasty, and in group B injection of 200 BT units into the pyloric sphincter muscle was used in 30 patients. An isotope-scan three weeks after surgery showed that five patients in group A and three in group B had delayed gastric emptying; there was no significant difference between the two groups, and the success rate of BT injection was 90% [115]. BT injection may be used instead of pyloroplasty as a simple, effective and complication-free method to prevent gastric emptying delay.

## 5. Duodenal and Biliary Applications

### 5.1. Sphincter of Oddi Dysfunction (SOD)

The sphincter of Oddi is a small ring of muscle that surrounds the biliary and pancreatic ducts just before they open into the duodenum. SOD is a poorly understood and controversial condition postulated to result in biliary pain, typically in the setting of a previous cholecystectomy. It has also been hypothesized that pancreatic SOD can result in pancreatic type pain and/or recurrent pancreatitis. The standard of SOD treatment currently is endoscopic sphincterotomy, which is a relatively high-risk procedure that is not uniformly effective. Hence there is interest in the use of a simpler procedure such as BT to serve as a therapeutic trial patients who respond to this treatment could then go on for more permanent relief using a sphincterotomy [132,133,134]. This was first suggested in a short report on two patients. Subsequently a larger study was reported evaluating twenty-two patients who had undergone cholecystectomy and had manometrically confirmed type III SOD [135]. Six weeks after 100 BT units injected into the sphincter, 12 patients (55%) were symptom-free, but ten patients (45%) were not. Of the ten patients who did not experience symptomatic benefit from BT injection, five had normal basal sphincter of Oddi pressures (<40 mmHg), and biliary sphincterotomy did not relieve the symptoms of these patients. Two of the remaining five patients with sustained sphincter hypertension after BT injection benefited from biliary sphincterotomy. Of the 12 patients who initially responded to BT injection, 11 patients remained symptom free for a median duration of six months. These patients had recurrence of biliary hypertension and responded to biliary sphincterotomy. The authors concluded that response to BT injection may select a subset of patients who will respond to biliary sphincterotomy.

BT has also been used with similar intent, although in an uncontrolled manner in patients with acute recurrent pancreatitis suspected to be due to pancreatic SOD [136].

The future role of BT injection in SOD still needs further investigation but current literature supports its use as a therapeutic trial in patients with SOD.

### 5.2. Others Biliary Disorders

BT induced relaxation of the sphincter of Oddi may help to treat patients with acalculous biliary pain [137]. A total of 11 patients had a positive response to BT injection of 100 UI into the sphincter of Oddi. Endoscopic biliary sphincterotomy has been induced a relief of biliary pain in 10 of these cases [137,138].

BT injection is as effective as endoscopic biliary stent placement in resolving cystic duct leaks in a canine model [139].

## 6. Pelvic and Anorectal Applications

### 6.1. Pelvic Floor Dyssenergia

Pelvic floor dyssenergia, also known as anismus, is a common cause of chronic constipation, hallmarked by inappropriate, paradoxical contraction or a failed relaxation of the puborectal muscle and EAS during defecation [2,3,140]. In normal patients, the puborectalis muscle and the EAS relax to straighten the anorectal angle and open the anal canal. Usually, this alteration in defecation is from maladaptive learning and responds to biofeedback in 60%–70% of patients as demonstrated in mostly single group, uncontrolled trials. Surgery has not been shown to be effective and has been largely discouraged as a treatment option. There are a limited number of studies evaluating the BT use in pelvic floor dyssenergia (Table 3).

**Table 3:** Published results of treatment of pelvic floor dyssenergia with BT.

Author	Pts	Name of Drug/Dose (units)	Results	Complication
Hallan *et al.*, 1988 [141]	7	Dysport—Nr	Maximum voluntary contraction from 70 to 28 cm H_2_O. Anorectal angle from 96° to 124°. Symptomatic improvement in four patients.	Incontinence in two patients
Joo *et al.*, 1996 [142]	4	Botox—6–15 U	Symptomatic improvement in all treated patients. Two patients relapsed.	0
Shafik *et al.*, 1998 [143]	15	Botox—25 U	Symptomatic improvement in 13 patients, on average 4, 8 months after the first treatment.	0
Maria *et al.*, 2000 [144]	4	Botox—30 U	75% were improved at 8 weeks. Anal tone during straining from 96.2 mm Hg to 42.5 mm Hg at 4 weeks, and to 63.2 mmHg at 8 weeks. Anorectal angle from 94° to 114°.	0
Maria *et al.*, 2001 [145]	14 AR	Botox—30 U	At 2-month evaluation, a symptomatic improvement was found in nine patients. At defecography, the rectocele depth was reduced from 4.3 ± 0.6 cm to 1.8 ± 0.5 (*p* < 0.001) and the rectocele area was reduced from 9.2 ± 1.2 cm^2^ to 2.8 ±1.6 cm^2^ (*p* < 0.001). The anorectal angle measured during straining increased from a mean of 98 ± 15° before treatment to a mean of 121° ± 19 ° (*p* = 0.001). At one-tear evaluation, there was no report of digitally rectal voiding and rectocele was no found at physical examination.	0
Ron *et al.*, 2001 [146]	25	Botox—20 U	Symptomatic improvement in 75% of the patients.	Perianal pain in 3 patients
Madalinski *et al.*, 2002 [147]	39	Botox—25 U	Nr	Perianal pain in 4 patients
		Dysport—150 U		
Albanese *et al.*, 2003 [148]	10 PD	Botox—100 U	Following treatment, anal tone during straining was reduced from 97.4 ± 19.6 mm Hg at baseline to 40.7 ± 11.5 mm Hg one month after treatment (*p* = 0.00001); no further change was observed at two-month evaluation (38.2 ± 10.4 mm Hg; *p* = 0.00001 *vs.* baseline values). The anorectal angle during straining (as measured with defecography) increased from a mean of 90° ± 7.9° before treatment to 122.2° ± 15° (*p* = 0.0004); nine patients evacuated the barium past without the need for laxative or enemas.	0
Cadeddu *et al.*, 2005 [149]	18 PD	Botox—100 U	At 2 months evaluation inspection revealed a symptomatic improvement in 10 patients. Anorectal manometry demonstrated decreased tone during straining from 96.2 ± 17.1 mm Hg to 45.9 ± 16.2 mm Hg at 1 month evaluation (*p* < 0.00001) and to 56.1 ± 10.7 mm Hg at 2 month (*p* < 0.00001). Pressure during straining was lower than resting anal pressure at the same times in all patients. Defecography after the treatment showed improvement in anorectal angle during straining witch increased from 99.1° ± 8.4° to 121.7° ± 12.7° at 2 months (*p* < 0.00001).	0
Maria *et al.*, 2006 [150]	24	Botox—60 U	At 2-month evaluation inspection revealed a symptomatic improvement in 19 patients. Anorectal manometry demonstrated decreased tone during straining from 98 ± 24 mm Hg to 56 ± 20 mmHg at 1 month evaluation (*p* < 0.01) and 56 ± 29 mm Hg at 2 months follow-up (*p* < 0.01). Defecography after the treatment showed improvement in anorectal angle during straining.	0
Keshtgar *et al.*, 2007 [151]	42	Botox—60 U	BT injection (*n* = 21) is equally effective and less invasive than M of IAS (*n* = 21) for chronic idiopathic constipation. At 3 months the median preoperative SS score improved from 34 to 20 in BT group (*p* < 0.001) and from 31 to 18 in the M group (*p* < 0.002). At 12 months the score was 19 and 14, 5 in BT and M group respectively (*p* < 0.0001).	0
Irani *et al.*, 2008 [152]	24	Botox—20 U	Of 24 patients, 22 experienced significant improvement in their constipation lasting greater than 22 weeks. There was a statistically significant improvement from 2.1 to 6.5 bowel movement per week (*p* < 0.001). The benefit of the BTX-A persisted a variable period of time among the responders, with 12 patient (55%) demonstrating a response lasting 6 months or more.	5 fecal soiling
Farid *et al.*, 2009 [153]	48	Dysport—100 U	In BFB group (*n* = 24) initial improvement was recorded in 12 patients (50%) while long-term success was recorded in 6 patients (25%). In the BT group (*n* = 24) clinical improvement was recorded in 17 patients (70.8%) but the improvement persisted only in 8 patients (33.3%). There is a significant difference between BT group and BFB group regard the initial success (*p* = 0.008) but this significant difference disappeared at the end of follow-up (*p* = 0.23).	Nr
Farid *et al.*, 2009 [154]	30	Dysport—100 U	BT injection (*n* = 15) achieved initial success in 13 patients (86.7%). Long-term success persisted only in six patients (40%). PDPR (*n* = 15) achieved initial success in all patients (100%) with a long-term success in ten patients (66.6%). However this difference did not produce any significant value. Recurrence was observed in seven patients (53.8%) and five patients (33.4%) following BT injection and PDPR, respectively.	0
Keshtgar *et al.*, 2009 [155]	16	Dysport—200 U	There were significant improvements in symptoms of constipation, soiling, painful defecation, general health and behavior, and fecal impaction of rectum (*p* < 0.05). Outcome was measured by a validated SS score questionnaire. At 3-months follow-up, the median SS score improved in all children after BT injection from 32.50 to 7.50 (*p* < 0.0001). At 12-months follow-up, the improvement of SS score in BT injection group was significantly more than the control group (*n* = 31) as follows: 4 *vs.* 15 respectively (*p* < 0.002).	0
Farid *et al.*, 2009 [156]	60	Dysport—100 U	The groups differed significantly regarding clinical improvement at 1 month [50% for BFB (n = 20), 75% BT injection (n = 20), and 95% for PDPR (n = 20), p = 0.006] and differences persisted at 1 year (30% for BFB, 35% BT injection, and 70% for PDPR, *p* = 0.02). BT injection seems to be successful for temporary treatment but PDPR is found to be an effective with lower morbidity in contrast to its higher success rate.	Nr
Ahmadi *et al.*, 2013 [157]	88	Dysport—160 U	Defecation of painful stool existed in 88% of patients before BT injection and it was reduced to 15% after BT injection (*p* = 0.0001). Stool was hard in 80% of patients before was reduced to 28% after BT injection (*p* = 0.0001). Soiling existed in 62% of patients before and was reduced to 8% after BT injection (*p* = 0.0001). Defecation intervals was 9.1 days, and after BTX-A injection was reduced to 2.6 days (*p* = 0.0001).	Nr
Zhang *et al.*, 2014 [158]	31	Xeomin—100 U	After treatment, the pressure of the anal canal during rest and defecation was significantly reduced from (93 ± 16.5) mmHg and (105 ± 28.3) mm Hg to (63 ± 8.6.3) mm Hg and (42 ± 8.9) mm Hg, respectively. BT injection combined with pelvic floor biofeedback training achieved success in 24 patients with 23 maintaining persistent satisfaction during a mean period of 8.4 months.	8 fecal incontinence

AR: Anterior rectocele; BFB: Biofeedback training; BT: Botulinum toxin; M: myectomy; Nr: Non reported; PD: Parkinson’s disease; PDPR: Partial division of puborectalis; SS score: Symptom severity score.

An initial trial evaluating seven patients with constipation and anismus received BT of unknown dose into the EAS [141]. Symptom scores improved significantly correlating with a reduction in the maximum voluntary and anal canal squeeze pressure and a significant increase in the anorectal angle on straining with subsequent fecal incontinence in two patients. In another study, with a sample size of 4 patients with anismus, the dose of BT ranged from 6 to 15 units injected into the EAS or puborectalis muscle under electromyography guidance [144]. All four patients, who had numerous failed biofeedback sessions, responded to BT with two patients having sustained responses for up to one year. A larger study evaluating 15 patients at a dose of 25 BT units injected into the EAS showed improvement in 13 patients (87%) for a mean of 4.8 months [143]. It is unclear whether BT should be injected into the EAS or the puborectalis muscle. Another study evaluated twenty-five patients who received 10 BT units on each side of the puborectalis muscle or 20 units in the posterior aspect of the muscle. Manometric relaxation was achieved after the first injection in 18 patients (75%), which endured throughout a six-month follow-up. Seven of 16 patients who failed the first injection had an additional one. Symptom improvement of 29.2% in straining index was recorded during follow-up with an overall satisfaction rate of 58.3%. Similar results have been noted in patients with Parkinson’s disease [149,159].

Rectoceles are commonly associated with outlet obstruction, such as pelvic floor dyssenergia. Therefore, decreasing anal sphincter tone during strain may decrease the size of the rectocele and improve symptoms of constipation. In a study of fourteen patients with anterior rectocele, each patient received 30 BT units at three sites, two on either side of the puborectalis muscle and the third in the anterior portion of the external anal sphincter, under ultrasonographic guidance [145]. At two months, nine of fourteen patients had symptomatic improvement with a decrease in rectocele depth and area and decrease tone during straining. At one year, no patient experienced incomplete or required digitally assisted rectal voiding.

BT use in the treatment of pelvic floor dyssenergia is still in its infancy with only small trials supporting its use. Many questions still remain such as the dose of BT, location of injection, use of ultrasound or electromyography, number of treatments, and combination with biofeedback. These questions need further study using placebo-controlled trials and larger sample sizes.

### 6.2. Chronic Idiopathic anal Pain

Chronic idiopathic anal pain is part of a rather ill-defined group of disorders termed chronic idiopathic perineal pain, which also includes proctalgia fugax and coccygodynia [160]. No objective abnormalities are found on clinical examination. The pathogenesis of the syndrome are unknown. There is no satisfactory treatment (anal stretch or surgery) for chronic anal pain. Eighteen patients who met the criteria for chronic idiopathic anal pain were studied. Treatment consisted of analgesics only in four patients, 0.2% nitroglycerin ointment in four, and ultrasound BT injection into the intersphincteric space in nine. Four patients were managed satisfactorily on analgesic treatment under the guidance of the hospital's pain clinic. Nitroglycerin ointment resulted in temporary pain relief in one of four patients. BT injection resulted in a permanent improvement in four patients, a temporary improvement in one patient, and no effect in four patients. Two patients had a colostomy, resulting in complete pain relief [160]. As in other syndromes based on muscular dystonia, some patients may benefit from BT injection.

### 6.3. Anal Fissure

Anal fissures are tears in the anoderm that start at the anal verge and can extend to the dentate line [161,162,163]. They can manifest into painful defecation and rectal bleeding. These fissures, which most commonly arise in the mid-posterior position of the anus, are thought to occur secondary to ischemia as a result of increased anal sphincter pressures and decreased blood flow [164,165]. Once chronic fissures develop, treatment options are aimed at interrupting this cycle by reducing sphincter tone using topical nitroglycerin, BT injection, oral nifedipine, or LIS performed surgically [165]. There are many reports on the efficacy of BT for this condition (Table 4).

**Table 4:** Comparison of published results on the treatment of patients with chronic anal fissure.

Author	Cases (*n*)			Units/injection’s Site	Healing rate (%)				Reinjection (%)/Dose	Complete Healing Rate (%)	Temporary Incontinence (%)	Recurrence (%)
					1 m			2 m				
Gui *et al.*, 1994 [166]	10			15 B/IAS	60			70	40/20 B	90	10	10
Jost *et al.*, 1994 [167]	12			5 B/EAS	Nr			83.3	-	83.3	0	8.3
Jost *et al.*, 1995 [168]	54			5 B/EAS	Nr			78	-	78	6	6
Jost 1997 [169]	100			2.5–5 B/EAS	Nr			82	-	82	7	8
Maria *et al.*, 1998 [6]	15			20 B/IAS	53.3			73.3	26.6/25 B	100	4	6.7
	15			Saline	13.3			13.3				
Maria *et al.*, 1998 [170]	23			15 B/IAS	21.7			43.5	8.7/20 B	100	0	0
	34			20 B/IAS	50			67.6	20.6/25 B	100		
Minguez *et al.*, 1999 [171]	23			10 B/IAS	48			Nr	52	83	0	37–52
	27			15 B/IAS	74				30	78		
	19			21 B/IAS	100				37	90		
Jost and Schrank, 1999 [172]	25			20 D/EAS	Nr			76	-	76	4	4
	25			40 D/EAS				80		80	12	8
Brisinda *et al.*, 1999 [173]	25			20 B/IAS	88			96	-	96	0	0
	25			0.2% GTN	40			60		60		
Fernandez *et al.* 1999 [174]	76			40 B/IAS	56			67	45.2/40 B	67	3	0
Maria *et al.*, 2000 [175]	25			20 B/IAS PI	48			60	24/25 B	80	0	0
	25			20 B/IAS AI	88			88	12/25 B	100		
Lysy *et al.*, 2001 [176]	15			20 B + ID/IAS	66			73	-	73	0	0
	15			20 B/IAS	20			60		60		
Madalinski *et al.*, 2001 [177]	14			25-50 B/EAS	Nr			54	-	54	0	8
Brisinda *et al.*, 2002 [178]	75			20 B/IAS	73			89	10.7/30 B	100	0	0
	75			30 B/IAS	87			96	4/50 B	100	3	4
Mentes *et al.*, 2003 [179]	61			20–30 B/IAS LIS	62.3			73.8	-	86.9	0	11.4
	50				82			98		98	16	0
Siproudhis *et al.*, 2003 [180]	22			100 D/IAS	50			32	Nr	NR	NR	NR
	22			Saline	45			32				
Brisinda *et al.*, 2004 [181]	50			50 B/IAS	82			92	-	92	22	0
	50			150 D/IAS	84			94	6/150 D	94	16	
Giral *et al.*, 2004 [182]	10			20 B/IAS	Nr			70	-	70	0	0
	11			LIS				82		82		
Simms *et al.*, 2004 [183]	47			30 B/IAS	Nr			Nr	17/Nr	78.7	0	27
Lindsey *et al.*, 2004 [184]	30			25 B/IAS + FIS	Nr			Nr	-	93	7	0
						1 m	2 m					
Arroyo *et al.*, 2005 [185]		40	25 B/IAS			Nr	85		-	45	5	55
		40	LIS				97.5			92.5	7.5	7.5
Arroyo *et al.*, 2005 [186]		100	25 B/IAS			-	88		-	47	6	53
De Nardi *et al.*, 2006 [187]		15	20 B/IAS			33.3	53.3		-	33.3	0	33
		15	0.2% GTN			13.3	66.7			40		33
Brisinda *et al.*, 2007 [188]		50	30B/90D/IAS			82	92		-	92	0	0
		50	0.2% GTN			58	70			46	0	34
Scholz *et al.*, 2007 [189]		40	10 B/IAS + FIS			95	Nr		5/Nr	79	2.5	10
Witte and Klaase, 2007 [190]		100	40–60 D/IAS			Nr	Nr		22/40–100 D	66	1	14
Festen *et al.*, 2009 [191]		37	20B/IAS + Poin			Nr	18.9		21.6/20 B	37.8	17.8	13.5
		36	1%ISDN + Pinj				44.4			58.3		25
Nasr *et al.*, 2010 [192]		40	20 B/IAS			55	62.5		-	62.5	0	40
		40	LIS			80	90			90	10	12.5
Samim *et al.*, 2012 [193]		60	20 B/IAS			25	43		-	32	5.5	11.7
		74	2% Dz			14	43			26		17.6
Valizadeh *et al.*, 2012 [194]		25	50 B/IAS			28	44		Nr	48	12	50
		25	LIS			40	88			92	48	8
Berkel *et al.*, 2014 [195]		27	60 D/IAS			Nr	66.6		3.7/Nr	66.6	18.5	28
		33	1% ID				33.3			33.3	12	50
Halahakoon *et al.*, 2014 [196]		30	40 B/IAS + AF			86.7	Nr		-	60	3.3	NR
Farouk, 2014 [197]		141	100 B/IAS + FIS			Nr	Nr		14/Nr	76	8	18
Gandomkar *et al.*, 2015 [198]		49	150D/IAS + 2%Dz			46.9	67.3		-	65.3	2	10.2
		50	LIS			74	92			94	7	0

AI: Injection in anterior midline; AF: Advancement flap; B: Botox (trade name of the type A preparation manufactured by Allergan, CA, USA); D: Dysport (trade name of the type A preparation manufactured by IPSEN, Maidenhead, UK); Dz: Diltiazem; EAS: External anal sphincter; FIS: Fissurectomy; GTN: Glyceryl trinitrate; IAS: Internal anal sphincter; ID: Isosorbide dinitrate; LIS: Lateral internal sphincterotomy; NB: Neuroblock (trade name of the type B preparation manufactured by Elan Pharma International Ltd., Ireland); Nr: Not reported; PI: injection in posterior midline; Pinj: Placebo injection; Poin: Placebo ointment.

These studies include several controlled trials comparing the toxin to either placebo or other modalities [6,170,173]. Clinical benefit is seen in the vast majority of patients, typically accompanied by reduction in resting anal sphincter pressure [175,178].

The exact site and dose of injection remains somewhat unsettled. Most of the trials to this point have evaluated BT administration at the point of the fissure, primarily, the posterior midline area of the anal verge. However, there is evidence that IAS fibrosis exists at the base of the fissure and is more prominent in this zone than other sites in the smooth muscle. This fibrosis may decrease the effects of BT on sphincter relaxation, thus delaying fissure healing. A study to evaluate this theory was conducted on 50 patients with posterior anal fissures who were either given 20 BT units lateral to the posterior fissure or 20 BT units on each side of the anterior midline [175]. After two months, a healing scar was observed in 15 patients (60%) of the posterior midline group and in 22 patients (88%) of the anterior midline group (*p* = 0.025). Resting anal pressure was significantly different from the baseline values at 1 and 2 months in both groups, but the values were significantly lower in patients of the anterior midline group.

Another study evaluated 150 patients with posterior anal fissures were treated with BT injected in the IAS on each side of the anterior midline. Patients were randomized to receive either 20 BT units and, if the fissure persisted, were retreated with 30 units, or 30 units and retreated with 50 units, if the fissure persisted [178]. One month after the injection, examinations revealed complete healing in 55 patients (73%) in the group receiving the lower dose and 65 patients (87%) in the group receiving the higher dose (*p* = 0.04). Five patients from the second group reported a mild incontinence of flatus that lasted 2 weeks after the treatment and disappeared spontaneously. The values of the resting anal pressure (*p* = 0.3) and the maximum voluntary pressure (*p* = 0.2) did not differ between the two groups. However, after two months, healing rates were similar between the two groups (89% and 96%). The authors concluded that the higher dose was more effective, but the improved effectiveness was not seen at two months [178].

The gold standard for treatment for anal fissures is surgery, primarily LIS. However, surgical intervention is associated with a low complication rate resulting in fecal incontinence, hematoma, and wound infection. A study compared BT injection (20 to 30 units) and LIS [179]. Overall healing rates were similar in both groups at six months with 10 of 61 patients requiring a second BT injection at two months. However, the response rate was higher at 1 and 2 months in the sphincterotomy group; 82% (41/50) at day 28 and 98% (49/50) at the second month (*p* = 0.023 and *p* < 0.0001, respectively, compared with the BT group). The response to BT was not as durable as surgery at 12 months falling to a success rate 75.4% (46/61) with seven recurrences in the BT group, whereas it remained stable in the LIS group (94%, *p* = 0.008). Sphincterotomy was associated with a significantly higher complication rate, eight cases of anal incontinence *versus* none in the BT group (*p* < 0.001) [179]. Thus, it appears that surgery is still the more durable treatment option but associated with more complications. These results have been supported in a more recent study. Some investigators have recommended surgery in younger patients and those with high resting anal pressures, as this is a risk factor for recurrence. Older patients may benefit from BT injection as they may be at higher risk of fecal incontinence.

A recent meta-analysis showed that even though LIS is associated with a better healing rate and recurrence rate, BT treatment is superior to LIS in overall complication rates and incontinence rates [199]. Thus, some advantages BT offers to patients with anal fissure include a good tolerance of the procedure, an outpatient setting, and a low risk of incontinence. The results of the meta-analysis are in line with previous research [200]. Furthermore, in a recent study BT injection was used not only as a therapeutic tool but also as a diagnostic test to identify patients who would not be suitable for further surgical LIS if they developed temporary incontinence after BT injection [201]. Combination therapy such as nitroglycerine and BT has also been evaluated; it appears that this only results in a modest increase in the rate of healing [202,203].

BT injection is efficacious in the treatment of chronic anal fissures. With greater than 60% response rates noted at two months with further response to re-treatment, BT can be considered a viable treatment option when more conservative treatment fails. In elderly patients, in who rates of fecal incontinence after surgery may be increased, BT can be considered first-line treatment. Surgery is still the most durable treatment option, but the risks of fecal incontinence must be weighed carefully against the benefits of the procedure.

Thus, according to many authors we recommend a safety first approach and treat all patients medically in the first instance. We believe that specific indications for surgical intervention in patients with anal fissure include persistence/recurrence, noncompliance or intolerance to the medical treatment. Patients at higher incontinence risk can be evaluated by anorectal manometric and endoanal sonography test, or, at best, the patient should be offered a sphincter-sparing procedure. The need for further investigations imposes a cost increase. Furthermore, it is difficult to calculate the increased cost in the event of complications. Some of these patients may wish to avoid LIS and persist with an alternative medical therapy.

Recently, Mishra *et al.* concluded that both treatments (NO donors and BT) may be considered as first-line treatment even if less effective than surgery [204]. However, this view has been challenged by other observations based on smaller series, providing inferior evidence of efficacy. The results of some studies are so disappointing that it led Nelson and coworkers to conclude a Cochrane review stating that “medical therapy for chronic anal fissure... may be applied with a chance of cure that is only marginally better than placebo.” [205]. We think that such conclusion is too pessimistic, and welcome further multi-center trials with appropriate methodology (intention-to-treat based selection of patients, doses, and injection technique) and adequate follow-up, to ascertain the safety and efficacy of the therapy. Moreover, the addition of multiple treatment modalities prolonged time to healing from initial evaluation, but allowed up to 75% of patients to avoid the need for permanent sphincter division while maintaining the highest rate of healing.

We believe that the introduction of conservative therapies, and especially of BT, in the treatment of these patients represents an innovation equal to the introduction of laparoscopy. The introduction of these therapies has made the treatment of anal fissure easier, in the outpatient setting, at a lower cost and without permanent complications. On the other hands, laparoscopy has led to an increase in the cost of a single surgical procedure, often with a higher incidence of complications than open surgery.

With regard to anal fissure, any conservative treatment used has lower costs than surgery [206]. Considering the three hypothetical scenarios reported in a recent paper, we found that the BT approach is more cost-effective than the ointment approach. In addition to cost reduction (on average 62% lower than the association NO donors plus surgery and on average 50% lower than the association CCA plus surgery), BT reduces the number of patients who need further surgery. Moreover, the preparation of incobotulinumtoxin A has a lower price than preparations onabotulinumtoxin A and abobotulinumtoxin A. This figure, given the similar clinical efficacy of the three formulations, would lead us to prefer the incobotulinumtoxin A [206]. It must be stressed, however, that the prices of the three formulations are not very dissimilar.

We believe that BT is a safe treatment for anal fissure; it should be considered the first-line therapy in patients with chronic anal fissure.

### 6.4. Other Anorectal Conditions

BT into the IAS has been applied both diagnostically and therapeutically after pull-though surgery for Hirschsprung’s disease. Minkes and Langer prospectively evaluated 18 such children who underwent BT injection (total dose 15–60 units) into 4 quadrants of the sphincter [207]. The authors have been showed improvement in 12 patients; improvement was sustained beyond 6 months in 5 of these patients.

A total of 33 children with surgically treated Hirschsprung’s disease treated with intrasphincteric BT injection for obstructive symptoms were analyzed in a recent study [208]. The median time of follow up was 7.3 years. A median of two injections were given. Initial improvement was achieved in 76%, with a median duration of 4.1 months. Proportion of children hospitalized for enterocolitis decreased after treatment from 19 to 7. A good long-term response was found in 49%. Basson and coworkers have been studied 43 patients with idiopathic constipation, Hirschsprung’s disease, anorectal malformation and GIT dysmotility [209]. A total dose of 200 BT units has been injected. Successful outcomes occurred in 72% patients after the first BT treatment, and 25% required further surgical management of their symptoms.

Pain after hemorrhoidectomy appears to be multifactorial; it seems be conceivable that IAS spasm is believed to play an important role [210]. The BT role in reducing pain after hemorrhoidectomy has been assessed in a double-blind study [211]. BT-treated patients have significantly less pain toward the end of the first week after surgery.

## 7. Conclusions

BT use for treatment of spastic GIT disorders has gained widespread acceptance over the last 15 years, especially in the treatment of chronic anal fissures and achalasia. Its administration is generally safe and relatively non-invasive compared to many of the alternatives. However, its short-term duration of action in disorders that affect patients long-term is its most significant negative. Repeated administrations with are generally necessary, with noted loss of efficacy.

The use of BT in many GIT disorders, although exciting, has not reached a level supported by clinical evidence. Further trials are needed with corresponding research to elucidate the pathophysiolgy of the spastic GIT disorders.
